# β-Caryophyllene Inhibits Endothelial Tube Formation by Modulating the Secretome of Hypoxic Lung Cancer Cells—Possible Role of VEGF Downregulation

**DOI:** 10.3390/ijms25020810

**Published:** 2024-01-09

**Authors:** Felix Wittig, Florian Koch, Liza Pannenberg, Sander Bekeschus, Robert Ramer, Burkhard Hinz

**Affiliations:** 1 Institute of Pharmacology and Toxicology, Rostock University Medical Center, Schillingallee 70, 18057 Rostock, Germany; felix.wittig@med.uni-rostock.de (F.W.);; 2 ZIK plasmatis, Leibniz Institute for Plasma Science and Technology (INP), Felix-Hausdorff-Str. 2, 17489 Greifswald, Germany

**Keywords:** β-caryophyllene, angiogenesis, CB_2_ cannabinoid receptor, vascular endothelial growth factor, lung cancer, hypoxia

## Abstract

β-Caryophyllene (BCP), a bicyclic sesquiterpene that is a component of the essential oils of various spice and food plants, has been described as a selective CB_2_ cannabinoid receptor agonist. In the present study, the effect of BCP on angiogenesis was investigated. It was found that conditioned media (CM) from BCP-treated hypoxic A549 lung cancer cells exhibited a concentration-dependent inhibitory effect on human umbilical vein endothelial cell (HUVEC) tube formation induced by CM from vehicle-treated hypoxic A549 cells. There was an associated concentration-dependent decrease in the proangiogenic factor vascular endothelial growth factor (VEGF) in the CM, with both BCP inhibitory effects (tube formation, VEGF secretion) being CB_2_ receptor-dependent. A reduction of the transcription factor hypoxia-inducible factor 1α (HIF-1α) was furthermore detected. The antiangiogenic and VEGF-lowering properties of BCP were confirmed when CM from another lung cancer cell line, H358, were tested. When directly exposed to HUVECs, BCP showed no significant effect on tube formation, but at 10 µM, impaired VEGF receptor 2 (VEGFR2) phosphorylation triggered by recombinant VEGF in a CB_2_ receptor-independent manner. In summary, BCP has a dual antiangiogenic effect on HUVECs, manifested in the inhibition of tube formation through modulation of the tumor cell secretome and additionally in the inhibition of VEGF-induced VEGFR2 activation. Because the CB_2_ agonist has no psychoactive properties, BCP should continue to be evaluated preclinically for further antitumor effects.

## 1. Introduction

The bicyclic sesquiterpene (*E*)-β-caryophyllene (BCP; synonyms: (-)-(*E*)-caryophyllene, (-)-β-caryophyllene, (-)-trans-caryophyllene) is a volatile compound that occurs in large amounts in the essential oils of various spice and food plants such as black pepper (*Piper nigrum* L.), oregano (*Origanum vulgare* L.), cinnamon (*Cinnamomum* spp.), and basil (*Ocimum* spp.) [1]. In addition, BCP was found to be a major component in the essential oil of *Cannabis sativa* L. [2]. Pharmacological interest in BCP has increased steadily since the discovery of its property to selectively target cannabinoid receptor 2 (CB_2_) as a full agonist with a *K*_i_ value of 155 nM [3]. In this context, the compound is particularly attractive because it possesses CB_2_ receptor agonist properties such as an anti-inflammatory action, but does not exhibit psychotropic effects typical of cannabinoid receptor 1 (CB_1_) activation (for review, see [4]).

In several studies, BCP has shown antitumor activity. Thus, an antiproliferative effect of BCP on human melanoma and breast cancer cells was described as early as the mid-1990s [5]. Later, anti-migratory and anti-invasive effects on colon cancer cells [6] and a proapoptotic effect on lymphoma cells [7] were demonstrated. In glioblastoma cells, both antiproliferative and proapoptotic properties of BCP were confirmed [8]. Moreover, several data suggest that BCP could be an attractive option for combined chemotherapies. Accordingly, BCP has been shown to enhance the growth inhibitory effects of paclitaxel in human breast cancer and colon cancer cells [9], to increase the anticancer properties of cisplatin against lung tumor cells [10], and to enhance the chemosensitization of human colorectal adenocarcinoma and leukemia cells to doxorubicin treatment [11].

However, to date, little is known about the effects of BCP on tumor neovascularization, whose inhibition is a major goal of cancer therapy. Angiogenesis is an important criterion for cancer growth and spread when solid tumors reach a diameter of more than 2–3 mm [12] and ensures the supply of oxygen and nutrients and the disposal of cellular debris. Under hypoxic conditions and nutrient deprivation, tumor cells secrete proangiogenic factors such as vascular endothelial growth factor (VEGF), which targets the receptor tyrosine kinase VEGF receptor 2 (VEGFR2) on the surface of endothelial cells to promote angiogenesis (for review, see [13]). Based on these findings, the VEGF antibody bevacizumab and several small molecule tyrosine kinase inhibitors (sunitinib, sorafenib, pazopanib) were developed and approved as part of current cancer therapies to combat tumor-induced neovascularization [14]. 

Regarding the effect of BCP on angiogenesis, a recent investigation observed decreased tumor neovascularization rates in both ectopic and orthotopic xenograft models after BCP treatment of mice [15]. In the same study, BCP was found to decrease human umbilical vein endothelial cell (HUVEC) migration, tube formation, and VEGF secretion and to inhibit vascular sprouting in the rat aortic ring assay. In addition, it decreased vessel density in the chorioallantoic membrane (CAM) assay [15]. On the other hand, there is no evidence of the antiangiogenic mechanisms of BCP in relation to the interaction between hypoxic cancer and endothelial cells. Currently, studies on the effect of BCP on cell behavior under hypoxic conditions are limited to a few investigations that focus on the effects on cerebral ischemic conditions [16] and hypoxia-induced neuroinflammatory responses [17].

In the present work, the impact of BCP on the tumor–endothelial interaction under hypoxic conditions was investigated. Using human non-small cell lung cancer (NSCLC) cells, it was found that conditioned media (CM) from BCP-treated hypoxic cells exhibited lower VEGF levels and caused decreased tube formation of HUVECs, with BCP exerting both effects in a CB_2_ receptor-dependent manner. In addition, BCP was shown to directly suppress VEGFR2 phosphorylation on endothelial cells independent of its CB_2_ receptor agonistic activity. These new findings argue for further elucidation of the mode of action of BCP as a potential antiangiogenic therapeutic option.

## 2. Results

### 2.1. CM of Hypoxic A549 Lung Cancer Cells Treated with BCP Inhibit Tube Formation of HUVECs

**Figure 1:**
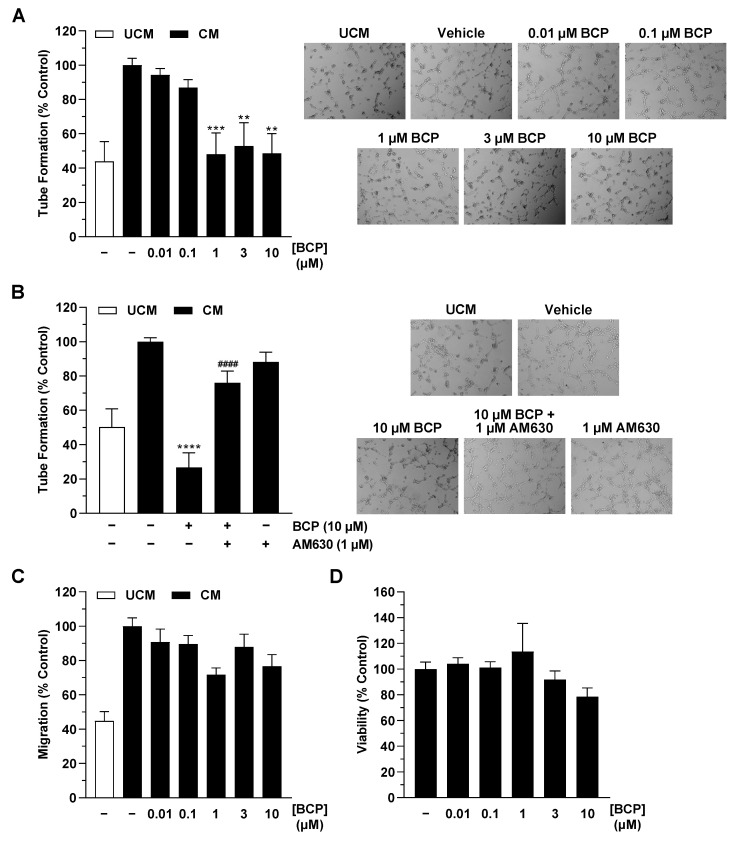
Effects of conditioned media (CM) obtained from hypoxic A549 cells treated with BCP on tube formation (**A**,**B**), migration (**C**), and viability (**D**) of HUVECs. The CM used were from hypoxic A549 cells previously incubated for 48 h with vehicle or the indicated concentrations of BCP. In the case of the experiment shown in (**B**), a 30 min preincubation with AM630 (1 µM) was performed under normoxic conditions. AM630 continued to be present in the incubate during the subsequent 48 h incubation with BCP under hypoxic conditions. Angiogenic features were determined after incubation of HUVECs with CM for 6 h (tube formation analysis) or 24 h (migration and viability assay). To the right of the bar graphs in (**A**,**B**) are representative light microscopy images of HUVECs from a tube formation assay after a 6 h incubation of HUVECs with UCM or CM of vehicle- and BCP-treated hypoxic A549 cells (50× magnification, Primovert inverted microscope). All percentage values refer to CM from vehicle-treated hypoxic A549 cells set to 100%. Serum-free vehicle-treated DMEM (unconditioned medium, UCM) was included for comparison. Data represent mean ± SEM of *n* = 13 (**A**,**B**), *n* = 9 (**C**), or *n* = 10–12 (**D**) per group. ** *p* ≤ 0.01, *** *p* ≤ 0.001, **** *p* ≤ 0.0001 versus CM from vehicle-treated hypoxic A549 cells; #### *p* ≤ 0.0001 vs. CM of BCP-treated hypoxic A549 cells; one-way ANOVA with Dunnett’s (**A**,**C**,**D**) or Bonferroni’s (**B**) post hoc test.

Previously published data with cannabinoids [18] or endocannabinoid degradation inhibitors [19,20] have shown that the investigated compounds do not directly inhibit the angiogenic properties of HUVECs. Accordingly, BCP also showed no significant effect on tube formation in HUVECs (Supplementary Figure S1A). On the other hand, in the work we have published so far, good evidence of an antiangiogenic effect of the cannabinoid test substances used was found when HUVECs applied for angiogenesis tests were in contact with the medium of cancer cells previously incubated with the respective substances [18,19,20]. On this basis, in the present study, CM were also obtained from hypoxic A549 cells treated with vehicle or BCP for 48 h. The corresponding CM were then used to suspend HUVECs and for appropriate angiogenesis assays. In agreement with recently published data [20], a comparison of vehicle-treated medium without cells and vehicle-treated hypoxic cancer cells revealed that the secretome of hypoxic cancer cells per se leads to increased tube formation (Figure 1A,B). Significant, mostly complete inhibitions of tube formation induced by CM from hypoxic A549 cells compared with unconditioned medium (UCM) were registered when A549 cells were previously incubated with BCP in the concentration range of 1 to 10 µM (Figure 1A). In a corresponding control experiment, the addition of UCM incubated with BCP or vehicle for 48 h under hypoxic, cell-free conditions did not significantly influence HUVEC tube formation (Supplementary Figure S1B). Taken together, prior contact of BCP with cancer cells, with presumed alteration of the tumor cell secretome, appears to be essential for its observed antiangiogenic properties.

To find an involvement of the CB_2_ receptor described in the literature as a BCP target [3] in the antiangiogenic effect shown, experiments with the CB_2_ receptor antagonist AM630 were performed next. Here, preincubation of A549 cells with AM630 used at a CB_2_ receptor inhibitory concentration of 1 µM [18,19,20,21,22,23,24] significantly abrogated the inhibition of endothelial tube formation induced by CM from BCP-treated hypoxic A549 cells (Figure 1B). In further experiments, a hypoxia-induced increase could also be registered for HUVEC migration, another angiogenesis parameter (Figure 1C). However, BCP showed neither a clear concentration-dependent nor a statistically significant effect here (Figure 1C). Finally, a comparative analysis of the effect of CM of BCP-treated hypoxic A549 cells on HUVEC viability revealed no significant effect compared with CM of vehicle-treated hypoxic cancer cells (Figure 1D).

### 2.2. BCP Inhibits Hypoxia-Induced VEGF Expression in A549 Cells

**Figure 2:**
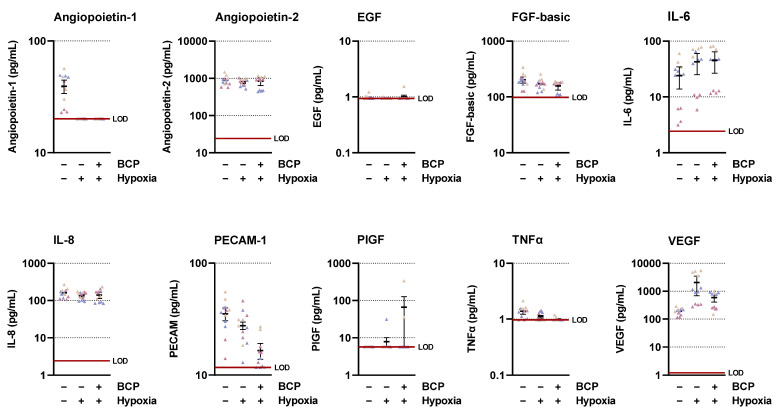
Concentrations of various angiogenic mediators in the CM of vehicle- or BCP-treated hypoxic A549 cells compared with vehicle-treated normoxic cells. The CM used were from hypoxic A549 cells previously incubated for 48 h with vehicle or BCP (10 µM). A vehicle-treated normoxic cell group was included to assess a potential hypoxia effect. Angiogenic mediators were determined in cell culture supernatants using the LEGENDplex™ multiplex assay. Each of the 3 independent experiments was measured in up to four technical replicates (colored coded triangles). The horizontal thick red line indicates the lower limit of detection (LOD) of the respective analyte. For a better visualization of the various effects, the y-axes are shown logarithmically. The thick short black horizontal lines in the diagrams represent the respective mean values ± SEM (thin black lines) of *n* = 3 independent experiments.

To find a possible mediator for the antiangiogenic effect of BCP, the regulation of various proangiogenic factors in hypoxic A549 cells was analyzed using the LEGENDplex™ multiplex assay (Figure 2). Here, hypoxia caused only upregulation of interleukin (IL)-6 and VEGF, whereas platelet endothelial cell adhesion molecule-1 (PECAM-1) and angiopoietin-1 were suppressed. No substantial change in secretion was found for angiopoietin-2, epidermal growth factor (EGF), fibroblast growth factor-basic (FGF-basic), IL-8, and tumor necrosis factor (TNF) α. In the case of the two upregulated factors, a downregulation of secretion could only be measured for VEGF. Furthermore, BCP led to a decrease in PECAM-1 levels, which, in contrast to VEGF, however, were not increased in the corresponding vehicle control compared with normoxia.

**Figure 3:**
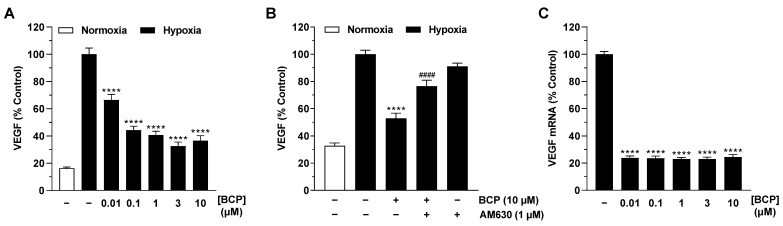
Effect of BCP on VEGF protein (**A**,**B**) and mRNA (**C**) expression in hypoxic A549 cells. A549 cells were incubated with vehicle or BCP under hypoxic conditions for 48 h (**A**,**B**) or 6 h (**C**). In the case of the experiment shown in (**B**), a 30 min preincubation with AM630 (1 µM) was performed under normoxic conditions. AM630 continued to be present in the incubate during the subsequent 48 h incubation with BCP under hypoxic conditions. All percentage values refer to vehicle-treated hypoxic A549 cells set to 100%. In (**A**,**B**), a vehicle-treated normoxic cell group was included to assess the hypoxia effect. Data represent mean ± SEM of *n* = 24 (**A**), *n* = 12 (**B**), or *n* = 6 (**C**) per group. **** *p* ≤ 0.0001 versus vehicle-treated hypoxic A549 cells; #### *p* ≤ 0.0001 vs. BCP-treated hypoxic A549 cells; one-way ANOVA with Dunnett′s (**A**,**C**) or Bonferroni′s (**B**) post hoc test.

Further analysis of hypoxia-induced VEGF expression was performed at the protein level with ELISA and at the mRNA level using RT-qPCR. Thereby, a strong induction of VEGF release from hypoxic A549 cells compared with normoxic conditions was shown at the protein level (Figure 3A). BCP, in turn, caused a concentration-dependent inhibition of hypoxia-induced VEGF protein levels in cell culture supernatant, which was significantly evident from as low as 0.01 µM (Figure 3A). Again, co-incubation with the CB_2_ receptor antagonist AM630 significantly abolished the BCP effect on VEGF formation (Figure 3B). Inhibition of VEGF expression by BCP was also registered at the mRNA level in A549 cells (Figure 3C). Interestingly, a significant effect was already observed at a BCP concentration of 0.01 µM, whereas a further increase in the concentration did not show any increase in the inhibitory effect.

### 2.3. BCP Decreases Hypoxia-Induced HIF-1α Protein Levels in A549 Cells

In a recently published work of our group, siRNA experiments demonstrated that hypoxia-induced VEGF protein formation in A549 cells proceeds independently of activation of the transcription factor HIF-1α [20]. In the case of BCP, however, we were interested in the regulation of this factor, which in addition to VEGF is responsible for the expression of other angiogenic factors (for review, see [25]) and genes involved in virtually all aspects of tumor biology, including invasion and metastasis, but also in the tumor′s response to radiation and chemotherapy (for review see [26,27,28,29]). 

**Figure 4:**
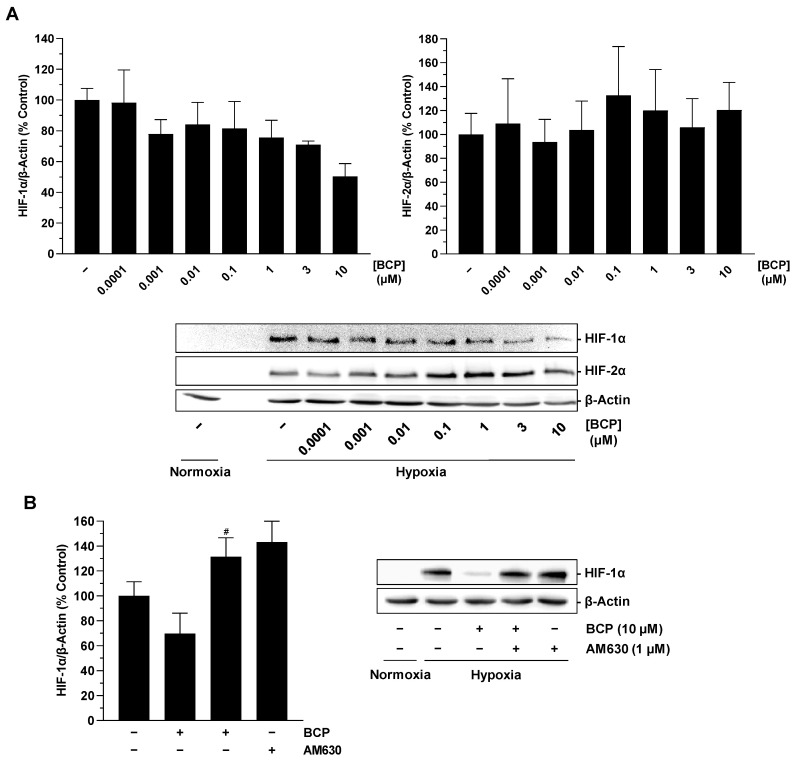
Effect of BCP on HIF-1α and HIF-2α protein levels in hypoxic A549 cells. A549 cells were incubated with vehicle or BCP at the indicated concentrations for 3 h under hypoxic conditions (**A**). In the case of the experiment shown in (**B**), a 30 min preincubation with AM630 (1 µM) was performed under normoxic conditions. AM630 continued to be present in the incubate during the subsequent 3 h incubation with BCP under hypoxic conditions. The Western blot images are representative of the experiments performed. A vehicle-treated normoxic cell group was included to assess the hypoxia effect (see line 1 of the blots shown). All percentages shown refer to vehicle-treated hypoxic A549 cells set at 100%. Data are mean ± SEM of *n* = 3 (**A**) or *n* = 4 (**B**) per group. # *p* ≤ 0.05 vs. BCP-treated hypoxic A549 cells; one-way ANOVA with Bonferroni′s post hoc test. In panel (**A**), a significant effect of BCP was excluded by one-way ANOVA with Dunnett′s post hoc test.

In the Western blot analyses we performed, BCP caused a reduction in HIF-1α in hypoxic A549 cells by approximately 30% and 50% after incubation with concentrations of 3 and 10 µM, respectively (Figure 4A, left). However, statistical significance could not be determined due to the high variability of the measured protein levels. Moreover, this inhibitory pattern did not correspond to VEGF regulation by BCP, where the latter elicited a highly significant VEGF decrease already at a concentration of 0.01 µM (Figure 3A). With respect to the transcription factor HIF-2α, virtually no interference by BCP was shown in the same experimental approaches (Figure 4A, right). In another experiment, the HIF-1α decrease detected in the presence of 10 µM BCP in hypoxic A549 cells proved to be reversible after preincubation with the CB_2_ receptor antagonist AM630 (Figure 4B).

### 2.4. BCP Also Inhibits VEGF Expression in Hypoxic H358 Lung Cancer Cells and Suppresses Endothelial Tube Formation via Modulation of the H358 Cell Secretome

**Figure 5:**
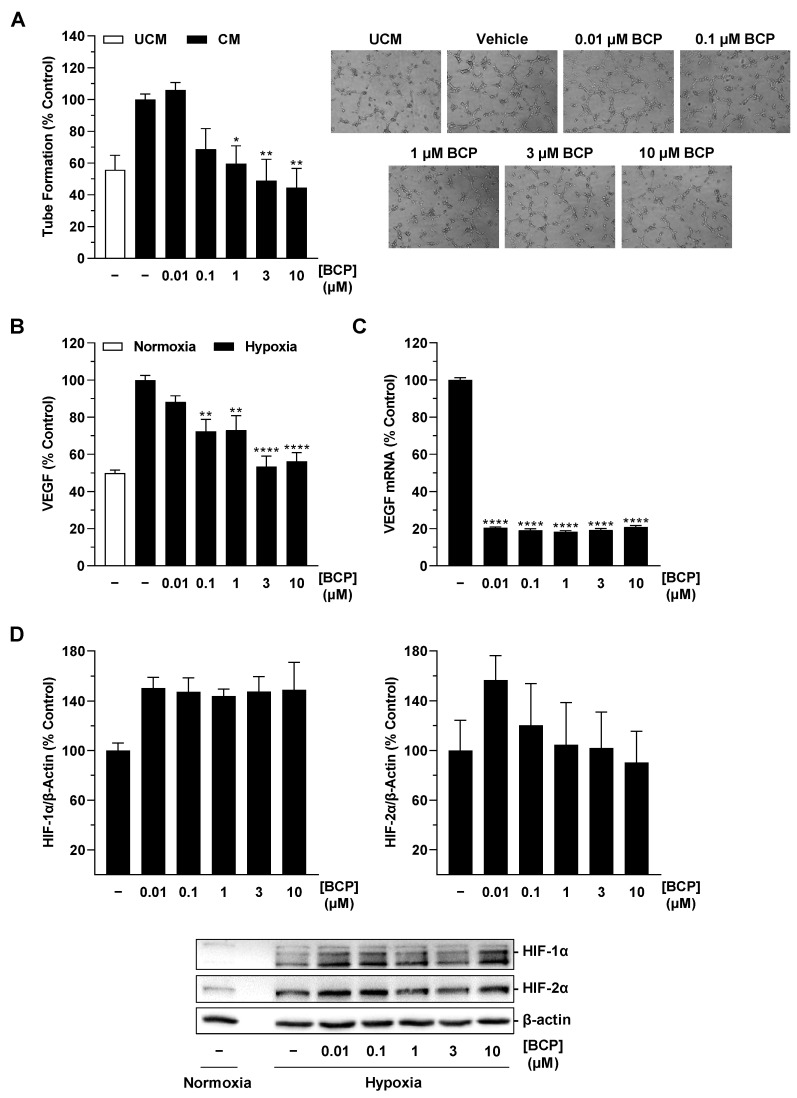
Effect of conditioned medium (CM) obtained from hypoxic H358 cells treated with BCP on tube formation of HUVECs (**A**) and of BCP on VEGF protein (**B**), VEGF mRNA (**C**), or HIF-1α and HIF-2α protein levels (**D**) in hypoxic A549 cells. The CM used (**A**) were from hypoxic H358 cells previously incubated for 48 h with vehicle or the indicated concentrations of BCP. In the other experiments, H358 cells were incubated with vehicle or BCP for 48 h (**B**), 6 h (**C**), or 3 h (**D**) under hypoxic conditions. To the right of the bar graph in (**A**) are representative light microscopy images of HUVECs from a tube formation assay after 6-h incubation of HUVECs with UCM or CM of vehicle- and BCP-treated hypoxic H358 cells (50× magnification, Primovert inverted microscope). Western blot images (**D**) are representative of each experiment. Percentage values given refer to vehicle-treated hypoxic H358 cells, set to 100%. Serum-free DMEM (unconditioned medium, UCM) (**A**) or vehicle-treated normoxic cells (**B** and Western blot images in **D**) were included for comparison (**A**) or to assess the hypoxia effect (**B**,**D**). Data represent mean ± SEM of *n* = 14 (**A**), *n* = 12 (**B**), *n* = 6 (**C**), or *n* = 3 (**D**) per group. * *p* ≤ 0.05, ** *p* ≤ 0.01, **** *p* ≤ 0.0001 versus vehicle-treated hypoxic H358 cells; one-way ANOVA with Dunnett′s post hoc test.

To exclude the possibility that the demonstrated antiangiogenic and VEGF-lowering effects of BCP were limited to A549 cells, key experiments were repeated with H358 cells, another NSCLC cell line. As shown in Figure 5A,B, BCP in H358 exhibited a similar pattern of effects as shown in A549 cells. Thus, concentration-dependent inhibition of HUVEC tube formation was also observed after incubation with CM of hypoxic H358 cells previously treated with BCP, which became statistically significant starting at a BCP concentration of 1 µM (Figure 5A). Similarly, BCP also caused a concentration-dependent inhibition of VEGF protein formation in hypoxic H358 cells, which was statistically significant at BCP concentrations between 0.1 and 10 µM (Figure 5B). In the case of the determined VEGF mRNA levels, again, a plateau effect in inhibition was noted starting at BCP concentrations of 0.01 µM (Figure 5C). On the other hand, the HIF-1α protein decrease previously registered in hypoxic A549 cells for higher BCP concentrations could not be confirmed in hypoxic H358 cells (Figure 5D). Interestingly, BCP, rather, led to an increase in HIF-1α protein levels here.

### 2.5. BCP Inhibits VEGFR2 Activation in HUVECs

**Figure 6:**
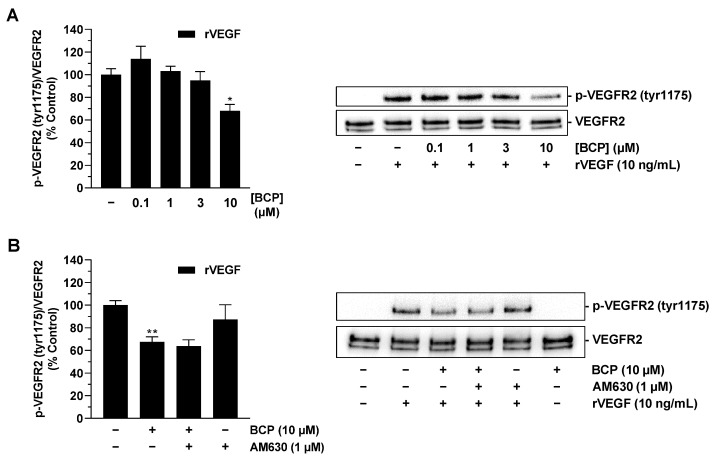
Effect of BCP on VEGF-induced VEGFR2 phosphorylation in HUVECs. HUVECs were preincubated with BCP at selected concentrations (**A**) or at 10 µM (**B**) for 6 h, followed by stimulation with recombinant VEGF (10 ng/mL) for 5 min. In the case of the inhibitor experiment shown in (**B**), AM630 was added to the cells 30 min before BCP. All percentages shown refer to vehicle-treated HUVECs set at 100%. Data are mean ± SEM of *n* = 3 (**A**) or *n* = 4 (**B**) per group. Western blot images are representative of each experiment. * *p* ≤ 0.05, ** *p* ≤ 0.01 versus vehicle-treated HUVECs; one-way ANOVA with Dunnett’s (**A**) or Bonferroni’s (**B**) post hoc test.

Having shown that BCP impairs the synthesis of VEGF in tumor cells, the final aim was to investigate whether BCP also impairs the effect of pre-existing VEGF. To this end, HUVECs were preincubated with vehicle or BCP and subsequently stimulated with recombinant VEGF. Activation of VEGF receptor 2 (VEGFR2), which has been described as a mediator of proangiogenic effects of VEGF in HUVECs [30,31,32], was investigated as a potential direct BCP target. This showed that preincubation of HUVECs with BCP resulted in significant inhibition of VEGF-induced VEGFR2 phosphorylation when the highest tested BCP concentration of 10 µM was used (Figure 6A). Interestingly, however, this was a CB_2_ receptor-independent BCP effect, as simultaneous treatment of cells with the CB_2_ receptor antagonist AM630 did not result in abrogation of the inhibitory effect of BCP (Figure 6B).

## 3. Discussion

Numerous studies in recent decades have shown that cannabinoids are a pharmacotherapeutic option that can inhibit tumor neovascularization. However, little is known about the mechanisms by which cannabinoids regulate tumor cell transactivation of endothelial cells, particularly under hypoxic conditions. Therefore, the present work provides unequivocal results suggesting that BCP causes alteration of the microenvironment of hypoxic lung cancer cells via activation of the CB_2_ receptor, which inhibits the tube formation of human endothelial cells associated with angiogenesis. Furthermore, a concentration of 10 µM BCP leads to additional direct CB_2_ receptor-independent inhibition of VEGFR2 activation of endothelial cells.

Several results suggest the above mechanisms. First, CM from BCP-treated hypoxic A549 lung cancer cells were shown to cause a concentration-dependent inhibition of HUVEC tube formation, which was associated with a likewise concentration-dependent decrease in hypoxia-induced VEGF levels. Thereby, direct exposure of HUVECs to BCP did not significantly alter the ability to form tubes, suggesting a specific effect of the A549 tumor cell secretome altered by BCP on the angiogenic properties of HUVECs. Second, the CB_2_ receptor antagonist AM630 abrogated both the antiangiogenic effect of CM from BCP-treated hypoxic A549 cells and the associated pattern of VEGF release. Third, the demonstration of functionally relevant key regulations in a second lung cancer cell line, H358, proved that this is not a cell line-specific effect. Finally, BCP resulted in inhibition of VEGF receptor activation in HUVECs treated with recombinant VEGF, which was not abolished by the CB_2_ receptor antagonist. 

Referring to the literature, BCP has also recently been shown to inhibit angiogenesis in the chicken embryo CAM model and in an orthotopic mouse tumor model, as well as in an ectopic model, using nude mice transplanted with a colorectal cancer cell line [15]. Here, BCP also exhibited antiproliferative properties against HUVECs with an IC_50_ of 41.6 µM, which, however, was far above the concentration range tested in the present report. Of note, the latter study showed a decrease in tube formation and migration of HUVECs in response to 5, 10, and 20 µM BCP, which is in contrast to our observation where tube formation of HUVECs treated directly with BCP was not significantly altered. The anti-tube-forming effects of BCP on endothelial cells in our hands were indeed limited to conditions of prior modulation of the tumor cell secretome. Interestingly, the significant inhibitory effect of CM of BCP-treated A549 cells on tube formation was not accompanied by a comparable inhibition of endothelial cell migration. Such selective modulation of angiogenic capacities has also been described by others for the effect of the vitamin E compound tocotrienol [33] and extracellular superoxide dismutase [34] on endothelial cells. As with our findings, the attack on the target site of new vessel formation, rather than the migration of cells to it, here, also appears to be the decisive factor in the antiangiogenic effect. Finally, it is noteworthy that antiangiogenic effects on HUVECs have also been described for aspfalcolide, a caryophyllene-type sesquiterpene lactone, although it is not clear whether this is a CB_2_ receptor agonist like BCP [35]. In addition, one study attributes a proangiogenic capacity to BCP. However, this study refers to BCP-containing plant extracts, as is the case with *Eugenia dysenterica* DC leaf essential oil, which contains 24.36% BCP and promotes angiogenic processes in the CAM test of chick embryos [36]. 

A functional contribution of the CB_2_ receptor to the antiangiogenic effect on endothelial cells has also been demonstrated by other authors. For example, impaired tumor vascularization of skin cancer xenografts, as determined by altered blood vessel morphology and decreased expression of proangiogenic factors (VEGF, placental growth factor (PlGF), angiopoietin-2), has been shown in nude mice treated with the CB_2_ receptor agonist JWH-133 [37]. Similar findings of decreased tumor tissue vascularization were observed after JWH-133 treatment of mice xenografted with glioma, astrocytoma [38], or melanoma cells [39]. In agreement with our results showing that BCP downregulates VEGF release and HIF-1α expression in lung cancer cells under hypoxic conditions, the same group had demonstrated by cDNA array analysis that JWH-133 reduces the mRNA of these angiogenesis factors in experimental gliomas [40].

Regarding the mechanisms of action triggered by CB_2_ receptor activation, a p42/44 mitogen-activated protein kinase-dependent inhibition of HUVEC migration was described in vitro [38]. However, other work argues against direct endothelial-driven CB_2_ receptor-initiated signaling pathways inhibiting angiogenesis. Accordingly, one study showed that HUVECs tended to exhibit slightly increased migration, tube formation, and 3-dimensional sprout formation after treatment with 3 µM JWH-133 without differing significantly from the vehicle control [18]. This is in partial agreement with the data presented here, where BCP at a concentration of 10 µM slightly increased tube formation when previously incubated for 48 h in medium under hypoxic culture conditions (UCM) but without contact with tumor cells. However, consistent with the presumed role of the CB_2_ receptor on cancer cells in an antiangiogenic effect mediated via modulation of the tumor cell secretome, CM of normoxic A549 cells treated with 3 µM JWH-133 resulted in a reduction in the angiogenic properties of HUVECs [18]. The underlying mechanism described here was an increased release of the antiangiogenic factor tissue inhibitor of metalloproteinase-1 (TIMP-1).

A further key finding of the present study is that the proangiogenic factor VEGF, which is induced under hypoxic conditions, is reduced under the influence of BCP. With respect to VEGF, it has been previously found that elevated VEGF levels alone are sufficient to promote angiogenesis [41], whereby VEGFR2 mediates the angiogenic effects of VEGF on HUVECs [30,31]. As shown in a recent paper, neutralization of VEGF in the CM of hypoxic A549 and H358 lung cancer cells resulted in significant inhibition of the otherwise stimulatory effect of the CM on VEGFR2 phosphorylation of HUVECs, confirming the critical role of VEGF in mediating the angiogenic capacities of these cells [20]. In this context, our finding that BCP attenuates the effect of VEGF at its cognate receptor beyond its effect on VEGF synthesis is very intriguing. This observation is consistent with the results of a computer-assisted structural analysis revealing potential BCP binding sites on the surface of VEGFR2 [15]. As in our study the inhibitory effect of BCP on VEGFR2 phosphorylation induced by recombinant VEGF was not altered by a CB_2_ receptor antagonist, the effect of BCP could indeed be explained by a direct interaction between BCP and VEGFR2. 

Another interesting result is the decrease in PECAM-1 in the supernatants of BCP-treated hypoxic A549 cells. However, the mechanisms of PECAM-1 in angiogenesis are unsettled and have led to conflicting results (for review, see [42]). Interestingly, previous work has nevertheless shown that PECAM-1 is involved in adhesion and/or signaling phenomena during angiogenesis that are required for endothelial cell motility and/or their subsequent organization into vascular tubes [43]. The precise role of BCP in reducing PECAM-1 in the supernatant of hypoxic A549 cells that we detected should, therefore, be clarified in future analyses.

An apparent discrepancy that stands out in this study is the difference between VEGF mRNA and protein levels after treatment with BCP. This contradiction is difficult to explain and suggests, on the one hand, a complex concentration-dependent BCP-induced downregulation of VEGF protein levels in the cell culture medium and, on the other hand, a mechanism that is already very efficient at low BCP concentrations at the mRNA level. However, such discrepancies between mRNA and protein levels are not uncommon in VEGF regulation. Accordingly, previous studies have demonstrated (i) VEGF regulations in which protein concentrations increase over time before mRNA concentrations [44], (ii) post-transcriptional regulation of VEGF concentrations in the cell culture supernatant, which are regulated via the membrane shedding of VEGF [45], or (iii) specific mechanisms that selectively regulate VEGF translation, resulting in significant differences between mRNA and protein regulation of VEGF [46]. Future investigations must show which mechanism triggers the functionally relevant concentration-dependent BCP-induced inhibition of VEGF concentrations in the cell culture medium. In any case, the data show that an mRNA analysis alone without protein measurements of VEGF is far from sufficient to describe a functionally relevant VEGF regulation.

Equally difficult to interpret is the role of HIF-1α in the regulation of VEGF release by BCP under hypoxia. Here, we found that in A549 cells, the reduction of HIF-1α correlated with the downregulation of VEGF, but this correlation was not detectable in H358 cells. However, recently published siRNA experiments revealed that both HIF-1α and HIF-2α do not play a critical role in hypoxia-induced VEGF secretion in A549 cells, whereas HIF-1α appears to mediate VEGF release from hypoxic H358 cells [20]. Accordingly, there is strong evidence that BCP-induced reduction in VEGF in hypoxic A549 cells is HIF-1α-independent despite the concomitant reduction of HIF-1α. Indeed, in addition to the studies demonstrating HIF-1α-dependent VEGF regulation, there is also work demonstrating HIF-1α-independent release of VEGF, as has been shown, for example, in human astrocytes exposed to pulsed electromagnetic fields [47], in colon carcinoma cells under normoxic conditions [48], or in astrocytes exposed to carbon monoxide [49]. In assessing the role and importance of HIF-1α in tumor growth, it should also be noted that in vivo experiments have shown that there are more effective pathways of proliferation that can override the pathways associated with HIF-1α signaling [50].

Overall, we have shown for the first time that BCP mediates an antiangiogenic effect via changes in the tumor cell microenvironment through CB_2_ receptor-driven VEGF inhibition, which may represent an attractive option for the treatment of tumors. Further studies are now needed to investigate the precise mechanisms by which BCP alters VEGF release under hypoxic conditions.

## 4. Materials and Methods

### 4.1. Materials

AM630 (#Cay10006974) was obtained from Cayman Chemical (Ann Arbor, MI, USA). Recombinant human VEGF-165 (rVEGF, #HZ-1038) and recombinant human serum albumin (rHSA, #HZ-3001) were purchased from Proteintech (Planegg-Martinsried, Germany). Dimethyl sulfoxide (DMSO), glycerin, glycine, sodium chloride (NaCl), sodium hydroxide (NaOH), hydrochloric acid 37% (HCl), Tris hydrochloride (Tris-HCl), and Tris ultrapure were purchased from AppliChem (Darmstadt, Germany). β-Mercaptoethanol was purchased from Ferak (Berlin, Germany). *Aqua ad iniectabilia* was bought from Braun Melsungen (Melsungen, Germany). (-)-trans-Caryophyllene (BCP; #22075), hydrogen peroxide solution (H_2_O_2_), luminol, bromophenol blue, *p*-coumaric acid, and paraformaldehyde (PFA) were purchased from Sigma-Aldrich (Taufkirchen, Germany). Acrylamide (Rotiphorese^®^ Gel 30), ammonium peroxydisulphate (APS), *N*,*N*,*N′*,*N′*-tetramethylethylenediamine (TEMED), and Tween^®^ 20 were purchased from Carl Roth (Karlsruhe, Germany). Dulbecco’s phosphate-buffered saline (DPBS) and fetal bovine serum (FBS) were purchased from PAN Biotech (Aidenbach, Germany). Non-fat milk (NFM) powder was obtained from Bio-Rad Laboratories (Munich, Germany). Gibco^TM^ Penicillin-Streptomycin (10,000 U/mL), Gibco^TM^ Trypsin-EDTA, and Gibco^TM^ Trypan Blue Solution were purchased from Thermo Fisher Scientific (Schwerte, Germany).

### 4.2. Cell Culture

Human NSCLC cells A549 were purchased from DSMZ (Deutsche Sammlung von Mikroorganismen und Zellkulturen GmbH, Braunschweig, Germany; #ACC-107; RRID: CVCL_0023). The NSCLC cell line NCl-H358 was obtained from ATCC (Manassas, VA, USA; #CRL-5807TM; RRID:CVCL_1559). Both lung cancer cell lines were maintained in Dulbecco’s modified Eagle medium (DMEM) with 4.5 g/L glucose and with UltraGlutamine^TM^ I from Lonza Cologne (Cologne, Germany) supplemented with 10% (*v*/*v*) heat-inactivated FBS, 100 U/mL penicillin and 100 μg/mL streptomycin. Human umbilical vein endothelial cells (HUVECs) were purchased from PromoCell as single-donor cryogenic vials (Heidelberg, Germany; #C-12200; RRID: CVCL_2959). HUVECs were cultured in Endothelial Cell Growth Medium Kit (#C-22110, assigned as HUVEC complete medium) from PromoCell supplemented with 100 U/mL penicillin and 100 μg/mL streptomycin. All cell lines were cultivated in a humidified incubator at 37 °C and 5% CO_2_. HUVECs were used between passages 3 and 7. The lung cancer cell lines were discarded after passage 20. 

For incubation with test compounds, cells were washed with DPBS and cultured in serum-free DMEM. In the case of viability studies with HUVECs, experiments were performed in serum-free DMEM with 1% (*v*/*v*) FBS. Test compounds were first dissolved in ethanol (BCP), DMSO (AM630), or DPBS (rVEGF) and then diluted in DPBS. The final concentration of solvents in the treated cell media was 0.1% (*v*/*v*) ethanol and/or DMSO and 0.000025% (*w*/*v*) rHSA. In all experiments, the incubation media contained the same amount of solvent. Experiments under hypoxic conditions were performed in the humidified Baker Ruskinn InvivO_2_^®^ 400 chamber (I&L Biosystems, Königswinter, Germany; hereafter referred to as hypoxia chamber) at 1% O_2_, 5% CO_2_, and 37 °C.

### 4.3. Generation of Conditioned Media from A549 and H358 Cells

To generate conditioned media (CM), A549 and H358 cells were seeded in 48-well plates at a density of 0.5 × 10^5^ cells per well and incubated for 16–24 h in a humidified incubator at 37 °C and 5% CO_2_ (normoxic conditions). Subsequently, cells were washed, and serum-free DMEM was added. For equilibration, cells were incubated in the hypoxic chamber for 15 min. After equilibration, incubation with BCP was performed in a final volume of 300 µL per well for 48 h in the hypoxia chamber. Similarly, unconditioned media (UCM) containing serum-free DMEM with the respective vehicle control without cells were prepared. The same procedure was used in the experiments with AM630, except that a 30-min preincubation with this compound was performed under normoxic conditions before hypoxia equilibration. Finally, UCM and CM were collected, centrifuged at 1300× *g* and 4 °C for 5 min, and used for HUVEC tube formation (Section 4.4), migration (Section 4.5), and viability (Section 4.6) analysis or LEGENDplex™ multiplex assay (Section 4.7) and VEGF analysis (Section 4.8). For some experiments, the media of a treatment group were pooled to obtain a larger volume.

### 4.4. Tube Formation Assay

The tube formation assay as a method to analyze the angiogenic potential of HUVECs was performed to determine a corresponding effect of CM of previously BCP- or vehicle-treated hypoxic A549 and H358 cells or of BCP and vehicle without prior tumor cell interaction. To this end, a previously described method [18,51] was applied with slight modifications. First, 48-well plates were coated with 30 µL of ice-cooled Corning^®^ Matrigel^®^ matrix (BD Biosciences, Heidelberg, Germany; #356234, hereafter referred to as Matrigel) per well, which polymerized at 37 °C for 30 min. Meanwhile, HUVECs were harvested and resuspended in serum-free DMEM. Then, the HUVECs suspension was seeded at a density of 0.5 × 10^5^ cells per well into a coated 48-well plate containing reoxygenated UCM/CM or serum-free DMEM with vehicle or BCP. After an incubation period of 6 h (in the case of UCM and CM) or 3 h (in the case of serum-free DMEM with vehicle or BCP) in a humidified incubator at 37 °C and 5% CO_2_, cells were fixed with 2% (*v*/*v*) PFA. Microscopic images were obtained using the Primovert inverted microscope (Carl Zeiss AG, Jena, Germany). To quantify tube formation, the number of tube-like structures that formed closed intersections was counted in four microscopic fields. 

### 4.5. Migration Assay

The effect of CM obtained from A549 cells on HUVEC migration was determined with a Boyden chamber assay using Falcon^®^ cell culture inserts (#353097; Corning, Corning, NY, USA) as previously described [18,51]. HUVECs were harvested and resuspended in reoxygenated UCM or CM from A549 cells (Section 4.3). Subsequently, 300 µL of HUVEC suspension containing 1 × 10^5^ cells were seeded onto inserts containing a polyethylene terephthalate membrane with 8 µm pores. DMEM containing 10% (*v*/*v*) FBS served as a chemoattractant in the lower companion plate. Non-migrating HUVECs on the upper membrane surface of the insert were removed with a cotton swab after an incubation period of 24 h in a humidified incubator at 37 °C and 5% CO_2_. Finally, the WST-1 assay was used to quantify the migrated cells on the membrane bottom.

### 4.6. WST-1 Assay

The WST-1 assay is a colorimetric test for the determination of cellular viability of HUVECs. In this assay, the water-soluble tetrazolium salt WST-1 (Roche Diagnostics, Mannheim, Germany) is cleaved by metabolically active cells to form a soluble formazan dye. In the corresponding experiments, after harvesting and resuspension in reoxygenated CM, 5 × 10^3^ HUVECs were seeded in 100 μL CM containing 1% (*v*/*v*) FBS per well of a 96-well plate. After an incubation period of 24 h, the metabolic activity of the cells was quantified by measuring the absorbance at 450 nm (wavelength correction at 690 nm) using a microplate reader (Infinite F200 Pro Tecan, Tecan Group, Männedorf, Switzerland).

### 4.7. LEGENDplex™ Multiplex Assay

CM of A549 cells were obtained as described in Section 4.3 and assayed for 10 key targets involved in angiogenesis using the LEGENDplex™ multiplex assay (BioLegend, Amsterdam, The Netherlands) according to the manufacturer’s instructions. The specific kit was the Human Angiogenesis Panel 1 (10-plex) with V-bottom plate (#740698). For the experiments, samples were incubated with capture beads with fluorescent barcodes coated with monoclonal antibodies against the following analytes: angiopoietin-1, angiopoietin-2, EGF, FGF-basic, IL-6, IL-8, PECAM-1, PlGF, VEGF, and TNFα. After washing, the beads were incubated with phycoerythrin (PE)-conjugated monoclonal antibodies specific for a different epitope of each analyte tested. After further washing, samples were detected by flow cytometry (CytoFLEX LX; Beckman-Coulter, Krefeld, Germany). The PE intensities thereby informed absolute analyte amounts by interpolation from 5-log analyte standards measured in parallel.

### 4.8. Determination of VEGF-A Protein

CM of A549 and H358 cells were obtained as described in Section 4.3. Subsequently, the VEGF Quantikine^®^ ELISA Kit (#DVE00) from R&D Systems (Wiesbaden, Germany) was used to quantify VEGF-A according to the manufacturer’s instructions.

### 4.9. Quantitative Reverse Transcriptase Polymerase Chain Reaction (RT-qPCR)

NSCLC cells were seeded in 6-well plates at a density of 2.0 × 10^5^ per well and maintained in DMEM containing 10% (*v*/*v*) FBS in a humidified incubator at 37 °C and 5% CO_2_ for 16–24 h. The cells were then washed, and serum-free DMEM was added under normoxic conditions. After equilibration in the hypoxia chamber for 15 min, cells were treated with BCP or its vehicle under hypoxic conditions. Following a 6 h incubation under hypoxic conditions, cells were lysed, and total RNA was isolated using the RNeasy Mini Kit from Qiagen (Hilden, Germany). Total RNA concentrations were measured using the NanoDrop™ OneC Microvolume UV-Vis spectrophotometer from Thermo Fisher Scientific to ensure the use of equal amounts of RNA for further RT-qPCR. The mRNA was quantified by RT-qPCR using the Applied Biosystems^®^ Taq-Man^®^ RNA-to-CT™ 1-Step Kit and primers for VEGF-A (Assay ID: Hs00900055_m1; FAM-MGB) and peptidylprolyl isomerase A (PPIA; Assay ID: Hs999904_m1; VIC-MGB) from Thermo Fisher Scientific according to the manufacturer’s instructions. PPIA served as a housekeeping gene to normalize VEGF mRNA levels before comparison with vehicle controls.

### 4.10. Isolation of Total Cellular Protein

NSCLC cells were seeded in 6-well plates at a density of 2.0 × 10^5^ per well and maintained in DMEM containing 10% (*v*/*v*) FBS in a humidified incubator at 37 °C and 5% CO_2_ for 16–24 h. The cells were then washed, and serum-free DMEM was added under normoxic conditions. For equilibration, cells were incubated in the hypoxic chamber for 15 min. After equilibration, cells were incubated with BCP or its vehicle in a final volume of 1 mL per well for 3 h in the hypoxic chamber. Similarly, normoxia control was prepared in a separate 6-well plate in a humidified incubator at 37 °C and 5% CO_2_. Cells were then washed and scraped in lysis buffer (2% [*w*/*v*] SDS, 40% [*v*/*v*] H_2_O, 10% [*v*/*v*] glycerol, 50% [*v*/*v*] 125 mM Tris-HCl [pH 6.8]). Proteins were immediately denatured at 95 °C with continuous shaking for 10 min. Subsequently, the lysed cells were centrifuged at 20,817× *g* and 4 °C for 5 min, and the supernatants were collected. 

For analysis of p-VEGFR2 and VEGFR2, HUVECs were seeded in 6-well plates at a density of 3.0 × 10^5^ cells per well and cultured for 16–24 h in HUVEC complete medium. After washing and cultivation in serum-free DMEM containing 10 µM BCP and, if necessary, 1 µM AM630, HUVECs were treated with 10 ng/mL rVEGF for 5 min. Protein isolation was then performed as described above. 

The Pierce™ BCA Protein Assay Kit (Thermo Fisher Scientific) was used to quantify protein concentrations. Before protein samples were stored at −20 °C, 4% (*v*/*v*) β-mercaptoethanol was added.

### 4.11. Western Blot Analysis

Equal amounts of protein samples were separated on an 8% SDS-polyacrylamide gel and transferred to a nitrocellulose membrane. Membranes were next blocked for 1 h in 5% (*w*/*v*) NFM in Tris-buffered saline containing 0.1% (*v*/*v*) Tween^®^ 20 (TBS-T buffer), washed in TBS-T buffer and incubated with primary antibodies in 5% (*w*/*v*) NFM in TBS-T buffer overnight at 4 °C. The primary antibodies used against VEGFR2 (#2479; RRID:AB_2212507) and p-VEGFR2 (Tyr1175) (#3770; RRID:AB_1642326) were purchased from Cell Signaling Technology (Frankfurt/Main, Germany). Antibodies against HIF-1α (#MA1-516; RRID:AB_325431; Thermo Fisher Scientific), HIF-2α (#NB100-122; RRID:AB_10000872; Novus Biologicals, Wiesbaden Nordenstadt, Germany), and β-actin (#A5441; RRID:AB_476744; Sigma-Aldrich) were used. Subsequently, membranes were washed with TBS-T buffer and incubated with secondary antibodies coupled to horseradish peroxidase (anti-rabbit antibody #7074, RRID:AB_2099233 or anti-mouse antibody: #7076; RRID:AB_330924) from Cell Signaling Technology in 5% (*w*/*v*) NFM in TBS-T buffer for 1 h at room temperature. A substrate chemiluminescence solution (100 mM Tris-HCl [pH 8.5], 1.25 mM luminol, 200 µM *p*-coumaric acid, 0.09% [*v*/*v*] H_2_O_2_) was then added, and signal detection was performed with the ChemiDoc XRS gel documentation system from Bio-Rad Laboratories (Munich, Germany). The Precision Plus Protein^TM^ Dual Colour Standard from Bio-Rad Laboratories was applied to identify the approximate molecular weight of the protein bands. The Quantity One 1-D analysis software (Bio-Rad Laboratories) was used to quantify the signal intensity of the protein bands. The specific protein bands were normalized to the signal of β-actin.

### 4.12. Statistics

All statistical analyses were performed using GraphPad Prism 9.4.1 (GraphPad Software, San Diego, CA, USA). Comparisons between two groups were performed using Student’s unpaired two-tailed *t*-test. Comparisons between more than two groups were performed using one-way ANOVA with Dunnett’s post hoc test when all conditions were compared with a vehicle control or using Bonferroni’s post hoc test for selected group comparisons.

## Data Availability

Data are available upon reasonable request from the first author.

## Supplementary Materials

The following supplementary information can be downloaded at https://www.mdpi.com/article/10.3390/ijms25020810/s1.
