# Supplementary material for: β-Caryophyllene Inhibits Endothelial Tube Formation by Modulating the Secretome of Hypoxic Lung Cancer Cells—Possible Role of VEGF Downregulation
Source: Int J Mol Sci. 2024 Jan 9;25(2):810. doi: 10.3390/ijms25020810 (PMC10815222; doi:10.3390/ijms25020810)
Supplement: Supplementary file 1 [file ijms-25-00810-s001.zip › ijms-2711984-supplementary.pdf]

International Journal of *Molecular Sciences*

Article

β-Caryophyllene Inhibits Endothelial Tube Formation by Modulating the Secretome of Hypoxic Lung Cancer Cells—Possible Role of VEGF Downregulation

Felix Wittig [1,†], Florian Koch [1,†], Liza Pannenberg [1], Sander Bekeschus [2], Robert Ramer [1] and Burkhard Hinz [1,*]

[1] Institute of Pharmacology and Toxicology, Rostock University Medical Center, Schillingallee 70, 18057 Rostock, Germany; felix.wittig@med.uni-rostock.de (F.W.)
[2] ZIK Plasmatis, Leibniz Institute for Plasma Science and Technology (INP), Felix-Hausdorff-Str. 2, 17489 Greifswald, Germany
[*] Correspondence: burkhard.hinz@med.uni-rostock.de; Tel.: +49-381-494-5770
[†] These authors contributed equally to this work.

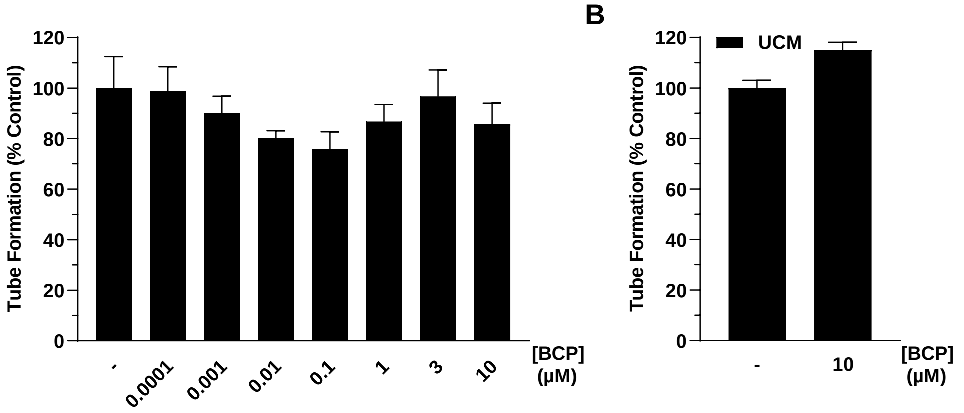

Supplementary Figure S1. Tube formation of HUVECs after direct incubation with BCP or vehicle (**A**) or with serum-free unconditioned medium (UCM) previously incubated with BCP or vehicle for 48 h under hypoxic, cell-free conditions (**B**). HUVEC were incubated for 3 h with (**A**) BCP at the indicated concentrations or vehicle in serum-free medium or with (**B**) UCM with vehicle or 10 µM BCP. Subsequently, the corresponding tubes were counted. Percentage values refer to the respective vehicle-treated control, set to 100%. Data represent mean ± SEM of $n = 3$. A significant effect of BCP was excluded by one-way ANOVA with Dunnett´s post hoc test (**A**) or Student´s unpaired two-tailed t-test (**B**).
